# Role of B Cells beyond Antibodies in HBV-Induced Oncogenesis: Fulminant Cancer in Common Variable Immunodeficiency—Clinical and Immunotransplant Implications with a Review of the Literature

**DOI:** 10.3390/diseases12050080

**Published:** 2024-04-23

**Authors:** Przemyslaw Zdziarski, Andrzej Gamian

**Affiliations:** 1 Lower Silesian Center for Cellular Transplantation, 53-439 Wroclaw, Poland; 2 Clinical Research Center PRION, 50-385 Wroclaw, Poland; 3 Hirszfeld Institute of Immunology and Experimental Therapy, 53-114 Wroclaw, Poland; andrzej.gamian@hirszfeld.pl

**Keywords:** IgG, protective level, vaccination, B cell, large granular lymphocytes (LGLs), common variable immunodeficiency (CVID), lymphocyte cooperation, hepatitis B immune globulins (HBIG), serum sickness, complement C4, hepatitis B virus (HBV), oncogenesis, hepatocellular carcinoma

## Abstract

Although lymphoma is the most frequent malignancy in common variable immunodeficiency (CVID), solid tumors, especially affected by oncogenic viruses, are not considered. Furthermore, in vitro genetic studies and cell cultures are not adequate for immune system and HBV interaction. We adopted a previously introduced clinical model of host–virus interaction (i.e., infectious process in immunodeficiency) for analysis of B cells and the specific IgG role (an observational study of a CVID patient who received intravenous immunoglobulin (IVIG). Suddenly, the patient deteriorated and a positive results of for HBs and HBV-DNA (369 × 10^6^ copies) were detected. Despite lamivudine therapy and IVIG escalation (from 0.3 to 0.4 g/kg), CT showed an 11 cm intrahepatic tumor (hepatocellular carcinoma). Anti-HBs were positive in time-lapse analysis (range 111–220 IU/mL). Replacement therapy intensification was complicated by an immune complex disease with renal failure. Fulminant HCC in CVID and the development of a tumor as the first sign is of interest. Unfortunately, treatment with hepatitis B immune globulins (HBIG) plays a major role in posttransplant maintenance therapy. Anti-HB substitution has not been proven to be effective, oncoprotective, nor safe. Therefore, immunosuppression in HBV-infected recipients should be carefully minimized, and patient selection more precise with the exclusion of HBV-positive donors. Our clinical model showed an HCC pathway with important humoral host factors, contrary to epidemiological/cohort studies highlighting risk factors only (e.g., chronic hepatitis). The lack of cell cooperation as well as B cell deficiency observed in CVID play a crucial role in high HBV replication, especially in carcinogenesis.

## 1. Introduction

Infections in humoral immunodeficiencies, including oncogenic viruses such as Hepatitis B virus (HBV), have a different clinical course. Research is focused on the coexistence of viral hepatitis and acquired immunodeficiency syndrome (AIDS) [1]. On the other hand, such primary or secondary immunodeficiency coexist with a high risk of malignancy. Unfortunately, most of the literature is devoted to B-cell lymphoma development in primary immunodeficiency (PID), especially EBV-related (for example, X-linked lymphoproliferative disease). Solid organ malignancy in PID is a new topic of interest. Furthermore, little is known about HBV virulence in posttransplant and primary immunodeficiency such as common variable immunodeficiency (CVID). Although the last cohort showed abnormal liver function and thrombocytopenia in most of the CVID patients, there is no standardized monitoring strategy for these patients. Evidence of liver disease was considered in CVID patients with abnormal liver function tests that should be repeated every 2–4 months, but there was no consensus on the frequency of abdominal ultrasound [2]. The tumor markers are not considered. The serology profile is difficult to interpret without HBV-DNA analysis [3]. For example, sometimes immune and liver abnormalities are observed, as described by Walter et al. [4]. An increase in total bilirubin was seen, but screening tests for HBV and HCV were negative. Although mortality associated with liver disease was noted in 63% of patients, and 7% of patients (3/38) had HBV, the HBV patients’ history and therapeutic regimen were not analyzed [2]. The cohort with comorbidities requiring immunomodulation did not describe solid tumors and cancer complications, in particular, leukemia/lymphoma only [2]. Notably, severe immunosuppressive therapy was used, for example, with rituximab (B-cell depletion) and anthracycline or alkylating agents, which have been associated with a potential risk for secondary malignancies [2]. Unfortunately, most hepatocellular carcinoma (HCC) cases are still diagnosed at an advanced stage, and this generally restricts the observational analysis of HBV-induced oncogenesis, the efficacy of therapies, and previous vaccination. Most studies on HCC immunosurveillance are focused on T lymphocytes and the microenvironment in advanced cancer [5]. These studies show potential therapeutic targets, but have an important limitation—they do not determine the cause, i.e., the initial stage of HCC development, but indicate the immune factors responsible for the progression (not oncogenesis) [5].

Furthermore, it is a paradigm that to provide sustained immune defense against virus replication, protective IgG must remain above a certain threshold level. Unfortunately, for most viral diseases, the protective titer is unknown [6]. For HBV, it is mainly derived from epidemiological studies based on vaccinated (immunocompetent) populations and public health. It is not surprising that there is no uniform agreement about the protective level of IgG against HBV surface antigen antibodies (anti-HBs). Following CDC, the patient is considered to be sensitive if the result is negative, i.e., <5 mIU/mL, >5.00 and <12.0 mIU/mL—indeterminate, and ≥12.0 mIU/mL—“positive” for anti-HBs, and the patient is immunized [7]. On the other hand, another CDC publication and the recommendations published previously by MMWR Recommendations and Reports indicate a much lower protection level—when greater than or equal to 10 mIU/mL [8] despite immunosuppression (e.g., drug-induced), the level of antibodies above the protective level is sufficient to avert exposure to the virus [9,10]. If anti-HBs decline (e.g., <10 mIU/mL in vaccinated persons) is sufficient to limit HBV replication [7] by the presence of a specific cellular immune response and expansion of B and T lymphocytes [11].

This common belief has been held by doctors in transplantology for years (e.g., after recipient vaccination after HSCT), especially in the case of liver transplants due to severe organ dysfunction, and also due to HBV, regardless of the serostatus of the donor and the recipient’s therapy [12]. Of note, transplant patients also present immunodeficiencies (iatrogenic), usually with severe lymphopenia. Furthermore, hepatitis B immune globulin (HBIG) is considered a sufficient defense against HBV, as it provides passive anti-HBs and temporary (i.e., 3–6 months) protection [7]. Clearly, this cannot be a good representation for immunodeficient patients and infants, who usually do not properly develop an immune response with a long IgG half-life and B cell memory, for example, in β2-Microglobulin deficiency [13]. The key issue is whether active or passive immunization is a sufficient protection against the genetic effects of HBV, and, therefore, HCC.

We introduced time-lapse analysis of standard observation and immunotherapy of CVID patients. Surprisingly, it demonstrated very fast infection, intensive HBV replication, and fulminant carcinogenesis, in spite of routine vaccination and passive immunization with a very high “protective” anti-HBs level.

Consent for the publication of clinical details was obtained from the patients in accordance with the Declaration of Helsinki. The local Bioethics Committee in decision No 13/24 has approved this publication and retrospective study (KB 32/24).

This observational study sheds light on the crucial role of the humoral compartment (beyond immunoglobulins) in HBV infection, as well as the low effectiveness and safety of specific IgG substitution in clinical immunology. This work expands on the previous model of antibody deficiency (described for CMV) in assessing its role in anti-HBV adoptive immunity [6].

## 2. Results

### 2.1. Initial Diagnosis

The CVID diagnosis was confirmed according to ESID criteria. Initial immunoparameters are presented in Table 1.

### 2.2. Results of Time-Lapse Analysis of the Patient’s History

**Figure 1:**
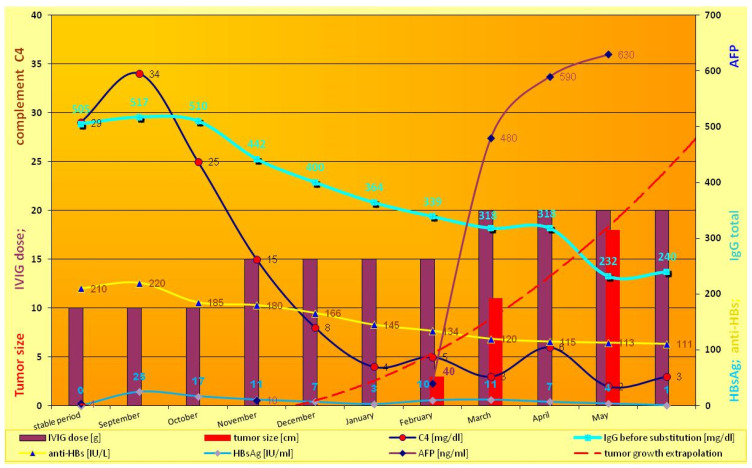
Severe and fatal HBV infectious process in a CVID patient despite IVIG therapy. Timeline shows HBV-induced hepatocellular carcinoma, serum sickness (with vasculitis and renal failure) development in spite of the “protective” anti-HBs level (yellow line). Very fast tumor growth corresponded with alfa fetoprotein (AFP) level. This indicates that the cancer process began around November (extrapolation based on available ultrasound data—dashed red line). The point corresponded with the start of a significant decrease in specific anti-HBs (to 185 mg/dL) within the period (September–November). The fall in the pound was exacerbated in spite of IVIG dose escalation. The individual parameters were distinguished by the colors and markers visible in the legend. The colors of the continuous line (measured values) correspond tothe colours in descriptions of the vertical axes. Extrapolated values (i.e., logarithmic growth of the tumor) are presented by a dashed line.

Antibody levels against HBV core antigens (anti-HBc total) were positive after IVIG therapy [3] (Table 1). Before subsequent substitutions, the level of IgG ranged from 505 to 1055 mg/dL, and anti-HBs from 111 to 220 IU/mL, depending on different preparations and series of IVIG and the patient’s compliance [3]. The baseline IgG level stabilized under the effect of substitution every four weeks and the patient functioned well (stable period, Table 1 and Figure 1). During this period, systematic diagnostic imaging (liver, spleen, lymph nodes) and biochemical tests were without deviations.

**Figure 2:**
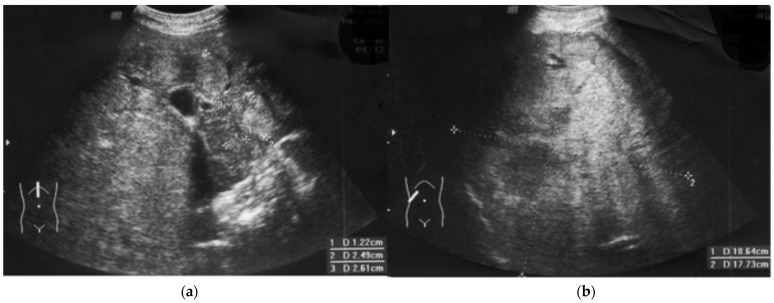
Fulminant hepatocellular carcinoma HCC progression in a patient with immunodeficiency. Regularly (every 3 months) performed ultrasound examinations (liver, spleen, lymph nodes) did not show any changes in October, but the ultrasound image showed heterogenous nodules (January), (**a**) that resemble nodular regenerative hyperplasia, typical for CVID [2]. The first blind liver biopsy (January) was non-diagnostic. Afterwards, a 110 mm tumor developed, and HCC was confirmed by second biopsy (April). Finally, fulminant growth up to 180 mm was observed despite lamivudine therapy, vaccination, normal ALT, and high anti-HBs level (**b**).

Suddenly and unexpectedly, the patient developed the following symptoms: muscle and joint pain, general weakness, lack of appetite, nausea and vomiting, and stomach pain. No jaundice was observed, and ALAT and bilirubin levels were normal. The family doctor recognized an intestinal infection. The patient received symptomatic treatment and diet. Microbiological tests were negative, but pruritus occurred. At the follow-up visit prior to subsequent IVIG substitution, the patient reported discolored stool and pain in the right hypochondrium (Figure 1, September). HBs antigen levels before substitution were positive, but the liver function test results were all normal (ALT = 33U/L, AST = 30 U/L). The pre-emptive lamivudine therapy [14], time-lapse analysis of anti-HBs, HBsAg, and complements C3 and C4 were introduced (Figure 1). He received 300 mg/d of lamivudine in divided doses. The level of HBV-DNA by polymerase chain reaction (PCR) was also examined. Real-time PCR showed extreme levels of viral load (61,500,000 IU/mL, i.e., 369 × 10^6^ copies). Opportunistic viruses (e.g., cytomegalovirus, Epstein–Barr virus) were negative, and HCV and HIV RNA were not detected. HDV-specific antibodies (class IgM and IgG) were negative during observation. Due to a significant decrease in the IgG level before the next substitution, the dose of IVIG was increased, although anti-HB levels were still high (Figure 1). Despite lamivudine therapy, lack of jaundice, rapid changes in mental status, or unexplained bleeding, the ultrasound image showed heterogeneous nodules (1.2–3 cm, see Figure 2a, i.e., from January).

The patient had progressive resistant thrombocytopenia (till 30–40 × 10^3^/μL) and coagulopathy (see below). At this stage (February), the patient was not qualified for surgery. Due to B cells < 1%, there was no target for rituximab. IVIG dose was increased for the treatment of thrombocytopenia in CVID [2] (Figure 1). Further on, computer tomography showed 11 cm intrahepatic tumor; afterwards, hepatocellular carcinoma and hepatic cirrhosis were confirmed by core needle biopsy. The first biopsy (January) was non-diagnostic and showed no significant abnormalities, whereas the second (April) (in stage IIIA-IVB HCC) was representative.

In blood analysis significant increase of AFP and HBsAg were observed (HbsAg fluctuation corresponded with further IVIG escalation) (Figure 1). Interestingly, a total and specific IgG-level decrease was observed. Subsequently, the patient developed vasculitis (Gianotti-crosti vasculitis), intensified with further IVIG dose escalation, that corresponded with C4 decrease (Figure 1), mixed coagulopathy, severe serum sickness, and acute renal failure (ARF). Therefore, the tomography was not performed. The complement C3 level was normal (therefore it is not presented in Figure 1).

### 2.3. Clinical Course of HCC and Outcome

Weight loss and significant deterioration (Eastern Cooperative Oncology Group (ECOG) status 4) were observed. Due to the patient’s condition, which included the risk of severe complications and immunodeficiency, the patient was disqualified from oncological therapy (e.g., immunotherapy with atezolizumab) by the Tumor Board [15]. Finally, metastases and hepatic encephalopathy were observed with fatal outcome within one month after HCC confirmation, and fast tumor progression was observed in ultrasonography (Figure 2b).

## 3. Discussion

### 3.1. Efficacy: Anti-HBs Immunogloibulins Are Not Protective Per Se

After the introduction of modern anti-HCV therapy, HBV-induced fulminant hepatitis and HCC are currently the most common causes of liver transplantation (LT). Patients with chronic HBV usually develop cirrhosis after several years of treatment-induced virological remission. Although the chronicity of HBV infection is attributed to inappropriate functioning of cell-mediated immunity, the humoral counterpart has not been analyzed. Our recent study indicates (using the example of CMV, i.e., common cause viral hepatitis in immunodeficient patients) that transient hipogammaglobulinemia in infancy as well as secondary (posttransplant) humoral immunodeficiencies may be a good model for clinical trials, i.e., patient-centered vaccination and monitored therapy [5]. Contrary to large-scale and statistically based studies on a potential infection and statistically estimated risk, these two models show real protection against an existing infection (i.e., CMV). The absence of signs of infection in even a very large group of immunized subjects (as in clinical trials of vaccines) does not mean protection, per se, because the actual exposure to the pathogen is unknown, only its certain probability. Accordingly, clinical and cohort studies on the effectiveness of specific hyperimmune immunoglobulins (HBIG) were conducted for a long time; their main shortcomings were a short duration and the general assumption of a protective titer > 100 IU [16]. Unfortunately, the systematic metanalysis of 2162 HBV-positive patients showed 6.6% recurrence regardless of the HBIG protocol [17]. In a single study, 14/147 patients (i.e., 9.5%) undergoing transplant due to HCC showed recurrence despite HBIG [18]. However, from the initial group, as many as 39 patients with HCC did not survive to the end of the study due to HCC, and two due to HBV-related complications. Furthermore, 27.5% of the donors were anti-HBs-positive, 20.1% were anti-HBc-positive, and, of note, 1.4% were HBsAg-positive. Therefore, about 7.4% of donors were potentially HBV-infectious. This corresponds with our current and previous observations of anti-HBc-positive immunoglobulins that are received from an extensive pool of blood donors (the tested preparations did not contain anti-HDV) [3]. Unfortunately, in transplant many years practice the HBV-positive (anti-HBs and anti-HBc total) donors are not disqualified. Despite high anti-HBs levels in 16 recipients receiving HBIG, one patient with residual anti-HBs titers below 50 UI/mL became HBsAg-positive [19], as in the presented case (Figure 1). The expression of IgG receptors (e.g., CD16) and complement concentration (classical cascade such as C4) significantly influence the IgG efficacy (see Section 3.2 and Section 3.3 below)

Unfortunately, the incidence of de novo HCC in the cohort of HBV-positive transplant patients has not been studied so far (“recurrence” only). In fact, we do not know how many patients with HCC “recurrence” relapsed from extrahepatic foci and how many developed HCC de novo due to HBV-mediated oncogenesis and post-transplant immunosuppression (see below). Our case here examined with a sudden onset of oncogenesis (fast tumor growth) and fulminant course indicates the latter scenario. It is the first signal for crucial B-cell role in HCC. Our observation is the first signal showing the role of mature B cells (in our patient in deep deficit—Table 1) in the protection against HBV-induced oncogenesis, independently of the antibodies. Unlike CMV, as we showed previously [6], the presence of HBV-specific antibodies in high titers do not ensure remission nor viral clearance: HBV, unlike CMV, does not form a virome. HBV latency (presence of HBV in hepatocyte without typical cycle) is beyond IgG and may be harmful.

### 3.2. HDV Probability

Typically, hepatitis D can be diagnosed serologically by identifying specific anti-HDV antibodies, IgM or IgG, in the examined person’s blood. Our patient was HDV-seronegative, but this issue has not been studied in CVID so far. It is extremely difficult because CVID incidence is low, and the number of HBV-infected subjects is a small fraction of CVID even in large multicenter studies [2]. Since HDV uses HBs, the possibility of HDV coinfection is unlikely: the patient was HBs-negative in routine time-lapse monitoring (anti-HBs antibodies may also have a protective effect). Of course, this possibility cannot be completely ruled out. On the other hand, HDV is associated with a greater likelihood of sequelae in the form of cirrhosis or liver failure, but not HCC, because HBV replication is inhibited in HBV/HDV coinfection. Of note, very high viral replication with viral load 369 × 10^6^ copies/mL was observed in the presented case.

### 3.3. Specific IgG Substitution Effectiveness and Safety

Liver transplantation (LT) in HBV-positive recipients, and especially in cases of HBV-induced defect, is the most controversial aspect of humoral protection and HBIG. The detectable HBV surface antigen level in spite of a concomitant increase in anti-HBs substitution indicates an overexpression of the HBV gene with very intensive HBs synthesis. It corresponds with immune-complex disease and significant classical complement cascade (Figure 1). Such a systemic reaction of antigen and antibodies, observed throughout the body, especially inside the vessels during substitution, was one of the elements of the deterioration of the patient’s condition (serum sickness). The reaction of HBs with specific antibodies resulted in the limitation of further mechanisms of response to the active virus in the infectious focus: classical complement cascade (Table 1, Figure 1). On the other hand, IgG limits the spread of HBV, but the local process (Figure 2) continued (see below).

ARF following prophylactic intravenous administration of HBIG was observed in a patient who had undergone a liver transplant. Such a reaction was described almost exclusively as an adverse effect of preparations containing saccharose [20]. What is noteworthy is that immune-mediated mechanism was not analyzed. On the contrary, intensive antigen–antibody reactions (Figure 1), and liver and lymphatic system damage with impaired elimination of immune complexes seem to be underestimated phenomena, especially in patients who have undergone splenectomy or liver transplantation. The liver contains the majority of C4 transcripts throughout the body [21]. Therefore, in hepatitis B and HBIG therapy, C4 seems to be a better and more universal signal of pathology than C3 and ALT levels, which were normal in our case.

A more relevant model, especially for clinical immunology and transplantation, is maternal–fetal transmission, with or without an HBV-passive mother and combined child prophylaxis [22]. In one study among 158 women, the intrauterine infection rate of newborns was 6.7%, and the chronic HBV rate of children was 4.0%. Although most mothers were treated for HBIG, five cases were HBsAg-positive. Interestingly, many infants did not appear to seroconvert after vaccination [23].

Moreover, in the original understanding, HBV pathology was associated with an immune reaction and not a cytopathic effect. Additionally, when immunity is distorted (as in our case), carcinogenesis begins to dominate.

### 3.4. Crucial Role of Lymphocyte Cooperation In Viral and Cancer Immunosurveillance

**Figure 3:**
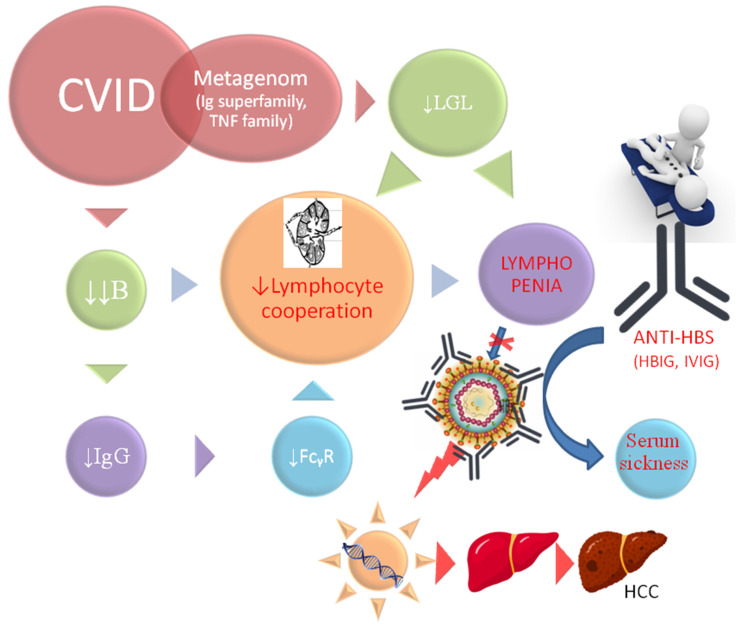
Crucial role of abnormal lymphocyte cooperation in HBV-induced oncogenesis. Immunoglobulin superfamily, (especially TNF family) genes, and corresponding protein expression are very low (e.g., IgG, IgA, IgM CD27, FcγR –Table 1). Therefore, abnormal cell–cell communication occurs. The levels and activity of large granular lymphocytes (LGLs) (e.g., expression CD16 receptor for IgG) are low. Lymphatic system atrophy causes lymphopenia due to low levels of innate (Nk), and adoptive lymphocytes (both Tc and B cells) (Table 1) cause a lack of effective lymphocyte cooperation. Immune synapse is defective and is downregulated in a metagenomic manner (as presented elsewhere [28] for monozygotic twins). Abnormal cell–cell communication results in impaired elimination of HBV, fast intracellular replication, easy integration into the host genome and, through genetic mechanisms, and fulminant oncogenesis. Intracellular mechanisms are beyond the action of IgG, and intense anti-HBs substitution (HBIG or IVIG) results in rapid serum sickness, intensified by poor elimination of immune complexes in atrophic lymphoid organs.

In immunocompetent patients, chronic inflammation precedes HBV-mediated oncogenesis. Recent studies have implicated T cells, especially CD8/Tc cells, but only in the HBV chronic process [24]. Tc cell activation and IFNα2 secretion may be crucial in acute inflammation [25]. However, since IFN-α2 was positively related to the frequency of CD8+ T lymphocytes in acute inflammation, the low level of these cells in our patient affected the innate immune response (Tc, B, and NK lymphopenia are observed—Table 1). On the contrary, viral clearance classically is monitored by serological conversion in patients infected with HBV (i.e., humoral and B-cell compartment). Viral clearance was difficult in our patient because it was associated with the HBV-specific adoptive immunity, which was damaged in CVID. The only indirect signs of a significant influence of B cells and humoral mechanisms on the fulminant course of HBV infection are the data from rituximab treatment, i.e., B-cell depletion therapy [14,26], which is overused in mild complications of CVID [2]. Interestingly, using the EUROclass system [27], our patient was classified into the group with nearly absent B cells (less than 1%); lymphatic atrophy and lymphopenia are among the key components of the class of CVID (Figure 3).

Although coagulation disorder was present in 79% of patients with the CVID-liver disease cohort, the immunosuppressive (B cell ablation therapy, cytostatics) was used for therapeutic regimen [2]. Vaccination and a high level of anti-HBs do not protect such patients from fulminant disease, because the lymphatic system is overloaded with immune complexes and lymphoma (treated with rituximab), and the B-cells are eliminated for long periods of time. Rituximab eliminates B cells (also B cell compartment in spleen and lymphatic system), causes antibody-dependent cell-mediated cytotoxicity (ADCC) with C4 complement consumption, and prompts a fulminant process, also without the presence of lymphoma [28]. From the perspective of our patient and CVID, the cooperation of B cells in the follicles seems crucial, since HBV replication and HBsAg secretion proceeded extremely dynamically (Figure 1), despite the passively maintained anti-HBs (in “protective titer”). The latest communication showed genetic and epigenetic mechanisms and abnormal immunity, and selected dysregulated interactions between B cells and the other immune cell compartments in the CVID [29]. A similar danger is posed by LT associated with deep immunosuppression, with anti-thymocyte globulin, anti-IL-2 receptor antibodies, as well as calcineurin inhibitors used in transplant procedures in 2.4%, 9.7% and 93.8% of patients, respectively [18].

The lack of specific immunoglobulin or immune response under the influence of antigen stimulation and B cells in our patient is very suggestive (Table 1). Interestingly, FcγRIII (CD16) is also underexpressed on lymphocytes (i.e., 5.2%—Table 1), particularly on large granular lymphocytes (LGLs) of both NK- and CD8-positive T-cells (i.e., 50.4 and about 17 cells/μL, respectively). Furthermore, this low expression may be a mechanism exacerbating the HBV-related state of CD8CD103-positive lymphocyte exhaustion in HCC [5]; therefore, this is a suggested here cause of the fulminant course. As ADCC activation with IgG/FcγRIII activation in LGL was damaged, one of the crucial mechanisms of passive immunization and antiviral immunity in our patient was significantly low. As in the T cells, NK cells induce IFNγ and TNFα gene transcription and cytokine mRNA. Interestingly, in NK cells, the signal is calcium-dependent and mediated by a cyclosporin A (CsA)-sensitive NFATp (the nuclear factor of activated T cells) [30]. CsA is, however, used in the first line of immunosuppression in transplant patients with HBV. The reduction in CD16 under the influence of CsA in transplant practice may cause a similar defect as in our patient with CVID. Adequate cellular compartment with positive delayed-type hypersensitivity in Mantoux tests and a normal CD4 level were not compensatory for the humoral and LGL defect. This is one of the problems with immunotherapy, which is a high risk for CVID patients, with unknown benefits (abnormal immune response). The lack of cause eradication (viral clearance and HBV oncogenesis) is significant in our case. Therefore, the incorporation of HBV-DNA into the host’s genome may initiate malignant transformation, even in the absence of chronic hepatitis or abnormal ALT. The viral genome incorporation and oncogenesis is beyond the action of IgG (Figure 3).

Although the level of CD8 T cells was reduced by about half to normal (i.e., 128 cells/μL), LGL T cells were significantly deficient among these cells, i.e., 17 cells/μL with clinical consequences (Figure 3). This is important information for the entire concept of low lymphocyte cooperation, deficiency of effector cells (i.e., LGL) with fulminant oncogenesis (presented in Figure 3). Thus, the observed changes in CVID gave a specific niche for the abundant multiplication of the virus and fulminant oncogenesis. HBx oncoprotein in complex humoral disorders, alongside the lymphatic system overload with immune complexes, caused HCC in many mechanisms in an uncontrolled manner and with fast growth (Figure 1) [31]. Both the proliferation associated with cyclins, MAPK, JAK/STAT3.5, and PI3K/NFkB signaling pathways and the disruption of apoptosis (p53), regardless of the increase in viral infectivity, are associated with intensive oncogenesis and death without cirrhosis in the observed case [32]. However, the initial and main cause is defective cell–cell communication and lymphoid atrophy, respectively.

Cellular (hepatocyte) transplantation may be a much safer option than non-selective harvest donors [33]. So far, despite the wealth of literature, oncological research has been focused on genetic issues or T cell compartment estimation in immunotherapy. The role of B cell immunosurveillance against oncogenic viruses is underestimated, especially against HBV in CVID [2,5].

Of note, immunoglobulin superfamily and TNF family gene expression are crucial regulators of B cell activation, usually modified in an epigenetic manner [28]. For example, transmembrane activator and calcium modulator and cyclophilin ligand interactor (TACI) mutation is frequently identified in CVID patients [34]. However, the earliest effect of TNF was discovered to inhibit tumor growth.

In the initial stage of HCC, nodular changes are similar to nodular regenerative hyperplasia (Figure 2a), treated with rituximab [2]. Malignancy development in patients with primary immunodeficiency requires a different investigative approach to immunotherapy [35].

Our observation, showing other humoral elements, including ADCC, LGL, and the complement cascade, is a good introduction to further observations and changes in existing immunotransplantation practice. Humoral immunity and complex B cell interaction cannot be replaced by passive immunization and the administration of specific immunoglobulins. Normal aminotransferase levels as satisfying results together with a lack of agreement on frequent imaging and HBV testing, as well as overlooking tumor markers, constitute a significant gap in the oncosurveillance against HCC developed in patient with immunodeficiency [2]. Our case demonstrates the need for fast surgical interventions and targeted biopsies. On the other hand, in our patient, other interventions such as surgery or chemotherapy with sorafenib were associated with profound risks, i.e., hemorrhage (including from the digestive tract, respiratory system, cerebral). The issue of assessing possible radical surgery is beyond the current manuscript, but transplantation (liver, HSCT) is questionable [15]. Unlike early HBV infection in mild CVID, i.e., the development of a chronic process, the risk of severe allergic reactions is much higher when the HBV infection occurs after IVIG initiation [3] (containing not only anti-HBs, but also anti-HBc IgG); moreover, C4 dynamics are more visible here (Figure 1). First of all, imaging diagnostics, e.g., regular ultrasound examinations of the liver (Figure 2), AFP (Figure 1) and early biopsy should be intensified, which is overlooked by some observations [36].

## 4. Materials and Methods

### 4.1. Material: Case Presentation

A 28-year-old, HBV-negative patient with common variable immunodeficiency (B-type) was qualified for intravenous IgG (IVIG) replacement therapy according to the ESID criteria [2]. The patient was not treated surgically and had not received any cosmetic procedures (including tattoos). He had no history of blood transfusion, alcohol consumption, nor injectable drug use. He used dental treatment only. In addition, he was vaccinated against HBV at birth and received a booster dose every 5 years. Initial immunoparameters are presented (Table 1). During the initial screening, the patient was negative for anti-HBs, HBc total, HBsAg, HBV-DNA, EBV, CMV, HCV, and HIV. Liver function tests and basic tumor markers (i.e., CEA, AFP, beta2-microglobulin) were normal. Checkup CT before IVIG starting (chest and abdominal) showed no abnormalities in the liver. No nodular lesions were observed in either the lungs or liver, i.e., sign of granulomatous–lymphocytic interstitial lung disease and nodular regenerative hyperplasia. Of note, the viral load in our patient was 61,500,000 IU/mL, contrary to 350,400 IU/mL described elsewhere [28,34]. Noteworthy, using the EUROclass system [27], our patient was classified into the group with B cells < 1% (Table 1). The patient gave written informed consent for this publication. The consent for publication of clinical details was obtained from the patients in accordance with the Declaration of Helsinki

### 4.2. Methods (Time-Lapse Analysis of CVID Presentation)

Regular (every 4 weeks) IgG replacement therapy was carried out with intravenous preparations at a dose of 0.3 g IVIG per kilogram of weight. The kinetics of real saturation were tested: serum IgG levels were determined (turbidimetry, Olympus) immediately prior to each IVIG infusion (Figure 1). Median IgG level was presented (Figure 1). Serum IgG and anti-HB levels significantly increased after immunoglobulin supplementation. The anti-HBs titer increased and was always over 100 IU/L (range 111–220 IU/mL), with the median level 210 IU/l as presented in the stable disease period (Figure 1).

Routinely, he had a chest X-ray and abdominal ultrasound (liver, spleen) and peripheral lymph node checks performed every 3–6 months, along with a chest CT every 12 months. Serum alanine aminotransferase (ALT) and bilirubin were tested every 3 substitutions. The peripheral blood lymphocyte profile was controlled annually by standard cytometry, EBV, and CMV with molecular techniques as a transplant patient in our center over a stable period [6].

## 5. Conclusions

Our case report is the first clinical signal for the low efficacy of IVIG/HBIG and the lack of “protection” with high anti-HB levels, with the key question of safety. Serum sickness, too-rarely diagnosed in the clinical background, is a key complication of IVIG/HBIG overuse, and a clear decrease in C4 (consumption or liver production) with AFP indicates a greater usefulness of these parameters than ALT. HBV-DNA (CVID patients receive frequent immunoglobulin injections), tumor markers and regular imaging (at short intervals) should be a daily practice in clinical immunology, especially in patients on B-cell depletion therapy or with hepatic involvement.

Our observation indicates one more key aspect. Even properly performed vaccination does not provide protection against HBV, if the patient has a deficiency of B cells, which are the primary antigen-presenting cells and are crucial for lymphocyte cooperation (Figure 3).

HBV infection, one of the strongest carcinogens, is one of the greatest challenges in immunological practice, in both primary and secondary (after rituximab) B-cell deficiencies. Apart from B-cell deficiency, abnormal lymphocyte cooperation in CVID, and the low CD8 level observed in this study, the expression of CD16 on LGL and ADCC is the second reason for atypical presentation with fulminant HBV-induced carcinogenesis. HBV-induced HCC in primary immunodeficiency is a very interesting and promising model for further research, but cancer development in such patients requires a different clinical and investigative approach to immunotherapy [36]. Simple treatment or chemotherapy is often dangerous (the risk–benefit ratio is imbalanced); despite the extension of progression-free survival (PFS), overall survival (OS) is shorter [15]. The treatment must be comprehensive (patient-centered care).

## Figures and Tables

**Table 1:** Basic immunoparameters and peripheral lymphocyte profile.

	*%lymphocytes*	*Cells/*μL	*Normal Range*
CD3	54.4	706.6	700–2100
CD4	42.7	555.5	300–1400
CD8	9.9	128.7	200–900
CD27+CD8+	7.9	102.2	
CD27+CD4+	40.2	522.1	
CD27+CD8+CD45 RO+	2.2	29.0	
CD27+CD8+CD45 RO-	5.6	73.2	
CD27+CD4+CD45 RO+	14.2	184.7	
CD27+CD4+CD45 RO-	26.0	337.4	
CD16	5.2	67.5	
CD56+CD3+	1.0	12.5	250–320
CD56+CD16+	3.9	50.4	90–600
CD19	0.9	11.2	100–500
CD27+CD19+	0.7	9.1	
**IVIG replacement therapy**	**Initial phase**	**Stable period**	**Normal range**
IgG [mg/dL]	80	505–1055	700–1600
IgA [mg/dL]	<5	NT	70–150
IgM [mg/dL]	<10	NT	40–230
Anti-HBs	<5 *	111–220	NA
Anti HBc total	Negative	Positive **	Negative
β2m (mg/L)	0.98	0.9–1.7	0.8–2.2
C4 [mg/dL]	38	29	10–40

* Anti-HBs as well as other vaccine-induced IgGs are not observed. Memory B cells and plasma cells in bone marrow were not detected. ** False positive, under the influence of passive transfer from IVIG [3].

## Data Availability

All data generated or analyzed during this study are included in this article.
